# Concealed Uterine Rupture in the Broad Ligament in an Unscarred Uterus With Postpartum Hemorrhage

**DOI:** 10.7759/cureus.26041

**Published:** 2022-06-17

**Authors:** Nicholas D Luke, Reynolds Risseeuw, Felipe Mercado

**Affiliations:** 1 School of Medicine, St. George’s University School of Medicine, True Blue, GRD; 2 Obstetrics and Gynecology, Trinitas Regional Medical Center, Elizabeth, USA

**Keywords:** postpartum hemorrhage, broad ligament, total abdominal hysterectomy, hemodynamic instability, spontaneous uterine rupture

## Abstract

Uterine rupture is an obstetric emergency that traditionally occurs during delivery in a mother with previously known risk factors, especially a uterine scar. However, a rupture that occurs in an unscarred uterus is uncommon. We report a case of a low-risk mother who presented for induction of labor for late-term pregnancy, without a history of uterine surgery, required vacuum-assisted vaginal delivery for maternal exhaustion, and one hour later was noted to have postpartum hemorrhage. The postpartum hemorrhage was managed medically but was taken for curettage due to continued hemorrhage and hemodynamic instability, followed by laparotomy which identified an 11 cm vertical uterine rupture parallel to the ascending uterine artery concealed in the broad ligament and extending inferiorly to the lateral cervix, necessitating a total abdominal hysterectomy. The patient tolerated the procedures well and was discharged home on postoperative day 2. Highlighting the importance of a high index of suspicion for uterine rupture, even without risk factors, this report describes an atypical presentation and describes an effective stepwise approach to management.

## Introduction

Uterine rupture is a life-threatening complication in the peripartum period, most frequently occurring with risk factors such as a prior uterine scar. Typical risk factors may include previous hysterotomy, use of induction/augmentation agents, high parity, obstetric maneuvers (including internal podalic version or the Kristeller Maneuver), overdistension of the uterine cavity, (multifetal gestations/macrosomia/polyhydramnios), and weak myometrium due to connective tissue disorders such as Ehlers Danlos type 4, placenta accreta, or abdominal trauma [1]. Uterine rupture is a rare occurrence, one study found a rupture incidence from 0.5 to 0.9% analyzing both history of one and two prior cesarean sections without statistically significant difference between them, even in the unscarred uterus, and a fetal mortality rate of 74-92% in affected individuals [1]. Other studies have shown perinatal mortality rates from 12 to 35% and rates of maternal hysterectomies from 20 to 31% [2,3]. While the incidence of unscarred uterine rupture is higher in developing counties, it should remain a differential for all obstetric practitioners. Describing this atypical presentation supplements the few reported cases existing in the current literature review.

With highly variable maternal and fetal presentation, uterine rupture may include maternal tachycardia, vaginal bleeding, constant/severe abdominal pain refractory to analgesics, loss of fetal station, as well as fetal bradycardia and/or variable or late decelerations [1,2]. Maternal and fetal outcomes depend on the identification of signs and symptoms and rapid surgical intervention given the often emergent nature of the pathology. We report a rare case of unscarred uterine rupture in the setting of a vacuum-assisted vaginal delivery and describe its presentation and management.

## Case presentation

In 2022 a 34-year-old woman, G3P2002 (the woman is pregnant with her third child and has two living full-term kids) presented to the labor and delivery department at 41 weeks and 0 days gestation age (GA) for induction of labor under midwife service. Her obstetric history included two full-term normal spontaneous vaginal deliveries in 2007 and 2015. Without any prior surgeries or procedures, her past medical history was significant only for herpes simplex virus II without recent outbreaks, and positive carrier status for alpha thalassemia and cystic fibrosis. The patient took 400 mg of acyclovir three times per day for 36 weeks GA. The patient denied any significant family/social/gynecological history. The patient had no lab or ultrasound abnormalities in her antepartum period.

On admission, the physical exam showed adequate pelvimetry, a category one fetal heart rate tracing that continued until the patient was in the second stage of labor. With irregular contractions, her cervical dilation was zero, effacement was at 30%, and the station was negative three with intact membranes. The estimated fetal weight by bedside ultrasonography was 4.108 g. Induction of labor began with a vaginal insertion of dinoprostone (10 mg) for 12 hours after admission, at which time cervical dilation was 2-3 cm, effacement 70%, and the station remained at −3.

**Figure 1:**
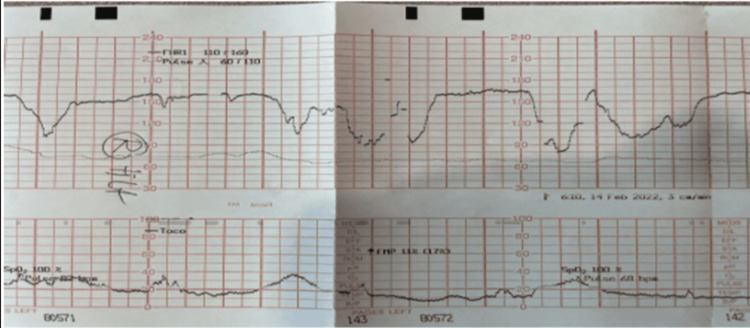
Fetal heart tracing 19 hours after admission showing category II tracing with late and variable decelerations.

**Figure 2:**
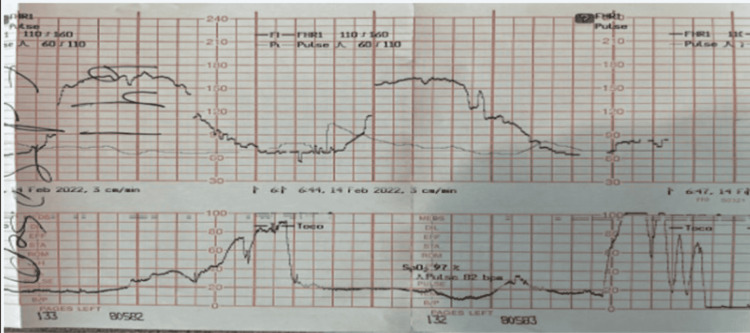
Category II fetal heart tracing at 20 hours post-admission with deep variables.

Low-dose oxytocin titration was begun at 2 mU/minute and titrated up to 2 mU/30 minutes until an adequate contraction pattern was achieved at a maximum rate of 4 mU/minute. Spontaneous rupture of membranes with clear amniotic fluid occurred 1.5 h after oxytocin initiation and an epidural was placed and found to be effective. The patient progressed to full dilation and +1 station after 5.5 h of oxytocin administration, maximum rate of 4 mU/minute with contractions every 2-3 minutes at which point the fetal heart tracing became category II-with minimal variability and recurrent deep variable decelerations and then recurrent late decelerations (Figures 1-2). Resuscitative measures including fluid bolus, maternal repositioning, and discontinuation of oxytocin were performed. The patient complained of severe pain with contractions, despite prior epidural bolus (vital signs stable), and subsequent maternal exhaustion with pushing.

Due to maternal exhaustion and worsening category II fetal heart tracing, the obstetric physician was called to perform a vacuum-assisted vaginal delivery which was uncomplicated and successful with one pull for 30 seconds. The fetus was delivered with APGAR (Appearance, Pulse, Grimace response, Activity, Respiration) scores of 8 and 8 at 1 and 5 minutes, respectively, after 19 h and 39 minutes, with the second stage of labor lasting 37 minutes. The placenta and membranes were intact and the fundus was firm, midline at the umbilicus. The perineum was intact and 1000 mcg of rectal misoprostol and 20 U IV oxytocin were given for bleeding prophylaxis. Quantitative blood loss (QBL) was 374 ccs.

Two hours after delivery a boggy lower uterine segment with a firm fundus was appreciated with active vaginal bleeding and postpartum hemorrhage was announced. The patient had a brief syncopal episode with a maternal heart rate of 120-150 bpm and blood pressure of 70/30 mmHg.

About 300 mL of urine was removed by a straight catheter. A 1-L lactated ringer solution was bolused, 10 U intramuscular Pitocin, 0.2 mg methylergonovine, and 1 g tranexamic acid were given with bimanual massage which expressed a small amount of placental membrane and a large amount of clot. Initially, blood pressure responded to 100/50 mmHg and a bedside transabdominal ultrasound was performed showing a small amount of contained fluid around the anterior lower uterine segment-suggestive of edema. The patient again became hypotensive and the decision was made to proceed with dilation and curettage in the main operating room. At this time blood loss was: 374 ccs from delivery, 1300 ccs from postpartum hemorrhage, and a total blood loss of 1674 ccs.

Under anesthesia in the main OR, 500 ccs of blood clots were extracted manually. Gentle banjo curettage was performed revealing minimal tissue/membranes. Metal suction curette was then used with a moderate amount of tissue extracted, at no point was there loss of resistance suggestive of uterine perforation. Suction curette was performed with a closed cervix after delivery and a ring forceps was used across the external os to maintain closed suction. At this time, 2 U of packed red blood cells had been transfused but there was continued heavy bleeding and the patient again became hypotensive and tachycardic. Given the continued bleeding and hemodynamic instability, with completed uterine evacuation, and physical exam significant for a firm fundus, equivocal for lower uterine segment atony, the decision was made to proceed with laparotomy. At this time blood loss was: 374 ccs from delivery, 1300 ccs on labor and delivery, 500 ccs during sharp and suction curettage for a total blood loss of 2174 ccs.

Low vertical midline abdominal entry was achieved and the survey was significant for a firm and intact appearing uterus with significant edema (consistent with prior bedside ultrasound), no ecchymosis, and no hemoperitoneum.

Uterine atony was excluded and no intraabdominal hemorrhage was appreciated in spite of continued vaginal hemorrhage and hemodynamic instability nonresponsive to fluid bolus, blood transfusion, medical management, and surgical evacuation of the uterus. The decision was made to forego O’leary and Haymann sutures, and instead proceed with supracervical hysterectomy.

**Figure 3:**
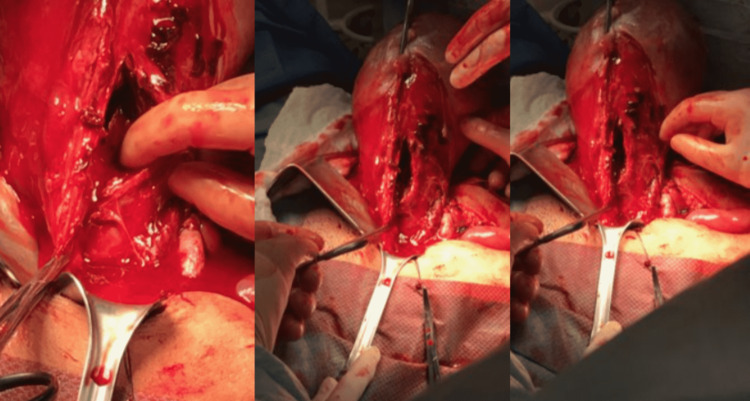
Exploratory laparotomy identified an 11 cm vertical uterine rupture parallel to the ascending uterine artery concealed in the broad ligament and extending inferiorly to the lateral cervix.

As the left-sided broad ligament was dissected inferiorly and bladder flap was created, a large 11 cm vertical rupture, concealed in the broad ligament and extending inferiorly to the lateral cervix was identified (Figure 3) that did not involve the uterine arteries. Notably, there was no subserosal hematoma identified in the broad ligament, suggesting the blood loss had flowed through the path of least resistance into the uterus and out of the cervix. Supracervical hysterectomy was completed but the vertical rupture line extended into the left superior cervix with continued bleeding and so a trachelectomy was performed to complete a total abdominal hysterectomy. Fallopian tubes were left in place due to significant adnexal edema. The case was concluded in a normal fashion. At the end of the case, blood loss during the hysterectomy accounted for 800 ccs for a total blood loss of 374 ccs from delivery, 1300 ccs on labor and delivery, and 500 ccs during sharp and suction curettage, 800 ccs from hysterectomy for a total blood loss of 2974 ccs.

The uterine and cervical pathology report described mostly blood and a few fragments of decidualized tissue in the endometrial curettage. The uterus had multiple 0.5 cm leiomyomata, and the endometrium expressed Arias-Stella reactions and decidualization.

## Discussion

Rupture of the uterus is an obstetric emergency that is usually linked to risk factors such as previous cesarean delivery that make the rupture more predictable to clinicians during delivery of the fetus. In the case of an unscarred uterine rupture, there are no known previous risk factors so the fetal and maternal mortality rates are very high [4]. The incidence of such events in developed countries is ~1:16,849 to 1:19,765, and it is theorized that the frequency of uterine rupture rates has decreased over time, despite increased rates of cesarean deliveries. This is likely due to unused obstetric practices such as internal podalic version and fundal pressure for labor dystocia [4]. In Nigeria, for example, this third-world nation has rates of uterine rupture that vary from 1:81 to 1:426 [5]. Poverty-stricken areas may have such high rates of rupture not only due to previous cesarean deliveries but also due to poor obstetric care, the use of traditional medical practices, ignorance, uterine anomalies, and poor infrastructure… [5]. Overall, data is very limited to unscarred uterine ruptures due to their rarity and the plethora of etiological factors that may influence uterine structural integrity.

Diagnosis of uterine rupture relies heavily on timing and imaging. Focused assessment with sonography in trauma (FAST) can be used in patients that are suspected to have uterine rupture based on presenting symptoms. Such symptoms include abrupt and severe abdominal pain, heavy bleeding, fetal bradycardia, loss of station, and uterine tenderness with cessation of contractions. Diagnosis can be made via laparotomy by visualization of complete disruption of all uterine layers with active bleeding and hemoperitoneum. The most common location of uterine ruptures is in the anterior lower uterine segment, however, the cervix, vagina, posterior uterus, and parametrium may be involved. Hysteroscopic and laparoscopic transillumination can be used concurrently to: accurately diagnose and locate the extent of the rupture, repair the rupture, and finally evaluate the uterine cavity (hysteroscopy) to assess for complete resolution of the defect [6].

In the case of our presenting patient, she received a total abdominal hysterectomy after the dilation and curettage failed to stop the postpartum hemorrhage. The dilation and curettage failed because there was no tissue within the uterus, only blood clots, this was an early sign to rule in a possible uterine rupture since the patient continued to bleed heavily after the dilation and curettage procedure. Due to hemodynamic instability, the patient received two units of packed red blood cells during the operation, one unit of fresh frozen plasma, two more units of packed red blood cells, and a complete blood count in the postanesthesia care unit. Postoperatively, the patient had recovered well and was sent home 2 days later. In the majority of uterine rupture cases, a hysterectomy is the treatment of choice, assuming the patient does not desire future pregnancy. Another treatment option is to close the laceration and leave the uterus intact, however, we could not perform this procedure on this patient since she was hemodynamically unstable and had a large cryptic laceration. Studies have shown that it is possible to repair uterine ruptures in about 90% of cases successfully, but the subsequent incidence rate was 4.3-19%, thus prompting clinicians to perform a cesarean section prior to the onset of uterine contractions [6,7]. Other treatment options can include the O’Leary suture, uterine artery embolization, and the use of Bakri or Jada balloon systems in order to control postpartum hemorrhage. In retrospect, the O’Leary suture and uterine artery embolization could have been performed at an early stage of intervention, but only if we had a high clinical index of suspicion for the occurrence of the uterine rupture. In this patient’s case, performing the O’Leary suture or the uterine artery embolization could pose a high risk of mortality in the mother due to current hemorrhaging. In regards to utilizing the Bakri or Jada balloon systems, these two options would give the illusion of controlling the bleeding in this patient, however, the blood would pool within the uterus and the patient would most likely die from hemorrhage. In general, using the balloon systems would be fatal if uterine rupture is suspected since bleeding is not stopped. Regardless of whether hysterectomy or suture repair is used, the most critical aspect of the survival of the patient relies on maintaining a high index of clinical suspicion, which allows us to act swiftly. Rapid surgical intervention is the gold standard, and it gives the mother and fetus the best chance at survival via decreased blood loss. If a uterine rupture is suspected or there is an unknown cause of abnormal vital signs then performing a manual exam of the uterine cavity is warranted. A manual examination can also shorten the time until diagnosis and thus lead to swifter management. Our patient did not receive a manual examination of the uterine cavity, which in retrospect delayed the surgical intervention and put the patient at a greater risk of mortality. A manual examination is crucial, but since we did not suspect the uterine rupture initially, it was overlooked and led to delayed treatment. In the future, a high index of suspicion should lead the clinician to perform a manual exam of the uterine cavity as early as possible in order to dictate the algorithm of management and treatment.

In our patient, it is also a possibility to consider an iatrogenic rupture, especially since a suction curette was involved. However, considering an iatrogenic rupture due to suction curettage would imply that several features of our presenting patient would be disregarded. First, there was a category II fetal heart rate at delivery with deep recurrent decelerations that suggests there that the uterus may have been compromised prior to initiation of suction curettage. Second, failure of maternal pushing at the end of the second stage of labor is likely explained by decreased uterine tone due to uterine rupture. Third, the patient became hemodynamically unstable postpartum and did not respond to initial conservative measures such as uterine massage. Next, the bedside ultrasound showed evidence of edema or subserosal free fluid surrounding the uterus. Finally, the bleeding continued immediately before, during, and at the completion of the dilation and curettage. While uterine rupture due to suction curettage is possible and should be kept on the differential, it is unlikely a cause of the bleeding in our patient. The rupture began during delivery and led to hemodynamic instability and decreased uterine tone prior to the dilation and curettage, making an iatrogenic cause less likely. In the future, preventing such scenarios of rupture is possible with cesarean sections and immediate hysterectomies if the patient does not desire future pregnancies.

In a study performed in Taiwan, reports of unscarred uterine rupture were recorded and they featured treatments and management that had some commonalities with our case. One particular case in the Taiwanese study described a case of uterine rupture with a delayed postpartum diagnosis which led to a hysterectomy and repair of a bladder rupture [8]. The delayed treatment of the rupture in the lower segment of the uterus consequently caused a bladder rupture to occur with an estimated blood loss of 4200 mL and complicated the obstetric emergency further [8]. This delay in surgical intervention is problematic and reinforces the need to maintain a high index of clinical suspicion amongst the medical staff. In another example in the Taiwanese study, a patient had a uterine rupture at the fundus, with an estimated blood loss of 800 mL, and received a transfusion, however, she did not have a hysterectomy; a common procedure to control the postpartum hemorrhage associated with uterine rupture [8]. The second Taiwanese case had a good maternal and fetal outcome, just like the first patient mentioned, however, she did not undergo a hysterectomy [8]. Even in the setting of an abrupt onset obstetric emergency, the surgeon was able to spare the reproductive organs and achieve outcomes comparable to the majority of patients that receive hysterectomies. Although her outcome was favorable, she would need to be carefully monitored in future pregnancies and will most likely undergo cesarean deliveries prior to active labor as a precaution.

In our patient, the fetal and maternal outcomes were very good and had no complications after the total abdominalhysterectomy (TAH). A stepwise approach was utilized to lead up to the TAH, in which conservative treatment was used first (uterine massage), followed by more drastic interventions (TAH). Another interesting feature of this uterine rupture is the discovery of the rupture postpartum, whereas traditional uterine ruptures are found during labor. Regardless of the temporal aspect of the rupture, the patient still left the hospital postoperatively on day 2 without complications. In one study, outcomes varied in the event of an unscarred rupture [9]. Eight ruptures were reported to have seven perinatal deaths (five stillbirths and two neonatal deaths) and two hysterectomies, but no maternal deaths [9].

Maintaining a high index of suspicion and treating the patient with rapid surgical intervention in a stepwise approach improves the survival rates of the mother and the fetus. In our patient, if the possibility of a rupture was identified earlier, it could have been possible to perform an O’Leary suture prior to the TAH. In theory, this could have saved her uterus for future pregnancies and hemodynamically stabilize her sooner. In retrospect, the O’Leary suture could not have been performed because the patient was hemodynamically unstable and the delayed discovery of the large and concealed laceration on the uterus would have led to even greater blood loss and possible maternal death. From this case report, we need to emphasize the importance of identifying early signs of low-risk unscarred uterine rupture in the antepartum and postpartum periods. Although rare in developed nations, this obstetric emergency still has a high incidence in developing nations that leads to future complications for the mother [5].

## Conclusions

Rupture of the unscarred uterus is a rare event in low-risk patients. The diagnosis is clinical and must be identified as early as possible due to its high maternal and fetal mortality rate. A stepwise approach is the best method to manage the rupture: starting with conservative treatment such as bimanual uterine massage and oxytocin augmentation, and then progressing to surgical interventions such as O’Leary sutures, uterine artery embolizations, and hysterectomies if all else fails. In our case, a total hysterectomy was required due to the unstable hemodynamic status of the patient, and the discovery of an 11 cm vertical uterine rupture parallel to the ascending uterine artery. This case report is intended to further increase awareness of uterine rupture, it is a low incidence but highly fatal obstetric emergency, especially if risk factors are not present. Treating this emergency requires a high index of clinical suspicion and must be approached in a stepwise manner to ensure an optimal prognosis.
